# Autologous Platelet Rich Plasma (PRGF) Preserves Genomic Stability of Gingival Fibroblasts and Alveolar Osteoblasts after Long-Term Cell Culture

**DOI:** 10.3390/dj10090173

**Published:** 2022-09-14

**Authors:** Eduardo Anitua, María de la Fuente, María Troya, Mar Zalduendo, Mohammad Hamdan Alkhraisat

**Affiliations:** BTI Biotechnology Institute, 01007 Vitoria, Spain

**Keywords:** platelet rich plasma, PRGF, cell therapy, genomic stability

## Abstract

Plasma rich in growth factors (PRGF) has several applications in dentistry that may require repeated applications of PRGF. Furthermore, it has been used for ex vivo expansion of human origin cells for their clinical application. One of the most relevant issues in these applications is to guarantee the genetic stability of cells. In this study, the chromosomal stability of gingival fibroblasts and alveolar osteoblasts after long-term culture was evaluated. Cells were expanded with PRGF or foetal bovine serum (FBS) as a culture medium supplement until passage 7 or 8 for gingival fibroblast or alveolar osteoblasts, respectively. A comparative genomic hybridization (CGH) array was used for the genetic stability study. This analysis was performed at passage 3 and after long-term culture with the corresponding culture medium supplements. The cell proliferative rate was superior after PRGF culture. Array CGH analysis of cells maintained with all the three supplements did not reveal the existence of alterations in copy number or genetic instability. The autologous PRGF technology preserves the genomic stability of cells and emerges as a safe substitute for FBS as a culture medium supplement for the clinical translation of cell therapy.

## 1. Introduction

Regenerative medicine has multiple potential applications and is geared towards the healing or substitution of damaged tissues to restore the previous function. The application of regenerative medicine in dentistry is motivated by the need of more personalized therapies and minimally invasive treatments [1,2,3]. Morbidity reduction and enhancing tissue healing (time and quality) would allow the clinicians to offer a better treatment to their patients.

Platelets and fibrin are two lifesaving molecules by supervising the hemostasia [4]. Many other functions have been attributed to the platelets as their implication in immunity, inflammation, and tissue homeostasis [5,6,7]. These fascinating living depots of biological clues have triggered the development of methods to enable their use in the clinical field [8,9]. Platelet rich plasma (PRP) refers to the fraction of plasma with platelet concentration above its concentration in the peripheral blood. For the preparation of PRP, venous blood is extracted in anticoagulant containing tubes that are subjected to centrifugation, separating the blood components according their gravity density [10]. The plasma column is then fractioned to obtain the platelet rich plasma. The reversion of the anticoagulant effect will activate the platelets and lead to the formation of fibrin clot [11]. Most of the studies concerning PRPs are devoted to the growth factors. These polypeptides allow for communication with the cells and modify their functions including cell proliferation, migration, differentiation, survival and metabolism [12]. Furthermore, fibrin exerts important biological functions by participating in the interaction between cells and matrix [13,14] and providing a scaffold that bridges a defect so that cell attachments and proliferation could take place [15,16]. Furthermore, fibrin switches the release of growth factors and cytokines (presents in plasma and platelets) from bolus kinetics to progressive controlled release kinetics [9,11,17].

Cell division is a survival tool that not only repairs an injury but also maintains the homeostasis. Platelet rich plasma has been shown to be mitogenic. In this sense, it is very important to preserve the integrity of the genomic material that are transferred to the daughter cells [18]. Cells have engineered molecular mechanisms (DNA damage checkpoint, DNA repair machinery and mitotic check point) that control the cell-cycle progression and the genome stability during cell division [18,19]. Increasing the risk of genomic instability would increase the chance of cell transformation toward malignancy [20,21]. Moreover, several clinical treatments may require repeated applications of plasma rich in growth factors as in the case of the management of oral lichen planus, mucous membrane pemphigoid, medication-related osteonecrosis of the jaw, oral and maxillofacial pain, and barrier-membrane defects [22,23,24,25,26,27,28,29].

Regarding cell therapies the utilization of ex vivo expanded human cells for clinical application is required. The complete absence of animal-origin derived products should be considered in order to avoid undesired reactions in the recipient [30,31,32].Regarding cell therapies the u. In this regard, foetal bovine serum (FBS) has traditionally been added to the culture media for cell isolation and in vitro expansion, thus being considered as the universal growth supplement; but potential risks of xenoimmunization and viral and zoonotic transmission exist when used for expanding cells destined for therapeutic purposes [33,34,35]. There is also a lack of standardization of FBS preparations with batch-to-batch variability, and an unsteady supply. Additionally, FBS has very low level of antibodies and contains higher concentrations of growth factors than calf and adult bovine serum. However, the procedure of obtaining FBS in slaughterhouses has raised ethical concerns due to the potential suffering of the calf fetus by the collection method [36]. These shortcomings and limitations establish the need for FBS-free technologies [37,38].

In this sense, the autologous therapy known as Plasma Rich in Growth Factors (PRGF) arises as a possible alternative to FBS supplementation in cell culturing. The biological potential of PRPs (platelet rich plasmas), specifically PRGF, has been highly demonstrated in different primary cells of different anatomical origins. PRGF has been shown to increase cellular functions (proliferation, migration, differentiation, and protein synthesis) and to have an anti-inflammatory effect [21,35,39,40,41]. Autologous PRGF gathers all the advantages of FBS as a culture medium supplement for cell isolation, maintenance, and propagation, thus avoiding the need of animal-origin components [35,42].

However, one of the most relevant issues is to guarantee the genetic stability of the expanded cells. For that purpose, in this study, the chromosomal stability of human gingival fibroblasts (hGFs) and alveolar osteoblasts (hABCs) after long-term culture with PRGF was evaluated.

## 2. Materials and Methods

This study was approved by Human Ethics Committee of the Hospital Universitario de Araba and performed following the principles of the Declaration of Helsinki, as revised in 2013. Primary cultures of human gingival fibroblasts and alveolar osteoblasts were established by the explant method as described before [43,44]. Written informed consent was obtained from all patients before the biopsy was performed. In the case of gingival tissue, samples were obtained from the maxillary tuberosity area of one male patient during routine surgical procedure. Tissue explants were incubated in Dulbecco’s modified Eagle’s medium (DMEM/F-12 (1:1 volume)) (Invitrogen, Grand Island, NY, USA) supplemented with 15% foetal bovine serum (FBS) (Biochrom, Berlin, Germany), 2 mM glutamine, 50 mg/mL gentamicin (both from Sigma-Aldrich, St. Louis, MO, USA). For alveolar osteoblasts isolation, sample was obtained during dental implant surgery. Tissue was explanted in osteoblasts culture medium (ObM) consisting in osteoblast basal medium with antibiotic complemented with osteoblast growth supplements (OGSs) and 15% FBS (all the reagents provided by Sciencell Research Laboratories, Carlsbad, CA, USA)). Explants from both origins were cultured in a humidified atmosphere at 37 °C with 5% CO_2_. When cells growing out from the explants reached sub-confluence, they were detached with animal origin-free trypsin-like enzyme (TrypLE select enzyme, Gibco-Invitrogen) and subcultured. Cell viability was assessed by trypan blue dye exclusion. Cells in passage 3 were used for the experiment. hGFs and hABCs were characterized by immunofluorescence as previously described [43,44]. Afterwards, the cell culture media were changed to DMEM/F12 + 10% FBS for hGFs and ObM + OGS + 5% FBS for hABCs for routine culture expansion. 

Blood from one young healthy female donor (37 years) was collected after informed consent into 9-mL tubes with 3.8% (*wt*/*vol*) sodium citrate. Samples were centrifuged at 580× *g* for 8 min at room temperature in system IV centrifuge (BTI Biotechnology Institute, S.L., Vitoria, Spain). Half of the tubes were used to obtain the whole plasma column (WP) over the buffy coat and the other half to take the immediately upper 2 millilitres over the buffy coat called Fraction 2 (F2). In both cases, care was taken to avoid the buffy coat containing the leukocytes. Platelet and leukocyte counts were performed with a haematology analyser (Micros 60; Horiba ABX, Montpelier, France). Both preparations were activated with 10% calcium chloride solution and the released supernatants were collected after centrifugation, filtered, and stored at −80 °C until use.

To determine if gingival fibroblasts and alveolar osteoblasts expanded with FBS, WP or F2 were genetically stable, both cell culture phenotypes at early and late passages were compared. Cells at passage 2 (hGFp2 and hABCp2) were thawed in multiwell culture plates with their corresponding culture medium at a density of 7000 cells/cm^2^. Cells collected after one passage were considered as starting point for CGH array analysis.

In addition, hGFp2 and hABCp2 were simultaneously cultured with DMEM/F12 + 10% FBS or 10% WP or 10% F2 and ObM + 5% FBS or 5% WP or 5% F2, respectively for further expansion. They were maintained in culture for several additional passages, allowing them to reach a high number of cell duplications (until passage 8 for hGFs and passage 7 for hABCs). Fresh culture medium replacement was carried out every 3–4 days. Cells were subcultured when the subconfluency stage was reached for each of the 3 culture conditions. After detaching, cell proliferation was measured in triplicate as population doubling level (PDL = 3.33 × log (total viable cells at harvest/total viable cells at seed)) using the trypan blue dye exclusion method.

Cells were collected and washed with Dulbecco’s phosphate buffered saline (D-PBS, Gibco-Invitrogen) at the starting (control) and final stages (expanded) of study. Finally, pelleted cells were maintained at −80 °C until the genetic analysis.

DNA extraction from the samples was performed using EZ1^®^ DNA Investigator Kit and EZ1 instrument. CGH array was performed using Cytoscan 750K arrays from Thermofisher (Waltham, MA, USA). Briefly, genomic DNA was digested, ligated and PCR-amplified. Then samples were purified, fragmented, and fluorescently labelled prior to sample hybridization in a Cytoscan 750K array. The array was finally scanned using a GeneChip^®^ 3000 Scanner (Thermofisher) and analysed with CHAS software (Affymetrix, Santa Clara, CA, USA) by comparing DNA from control and long-term expanded cells. Results fulfilling quality controls were finally analysed. For bioinformatics analysis, the recommendations of the manufacturer’s software were followed. A change in the copy number (copy number variations, CNVs) is reported for a minimum of 50 consecutive markers. The average resolution of the array is one marker every 1737 bp for intragenic regions and one every 6145 bp for the rest of the genome.

## 3. Results

### 3.1. Blood Cell and Platelet Counting in WP and F2 Preparations

Platelet enrichment of the PRGF-Endoret preparations was 2.05-fold for whole plasma (465 × 10^6^ platelets/mL) and 2.22-fold for Fraction 2 (428 × 10^6^ platelets/mL) over the baseline concentration in the whole blood (Table 1). None of the preparations contained noticeable concentrations of leucocytes or erythrocytes.

### 3.2. Gingival Fibroblasts and Alveolar Osteoblasts Cultures

**Figure 1:**
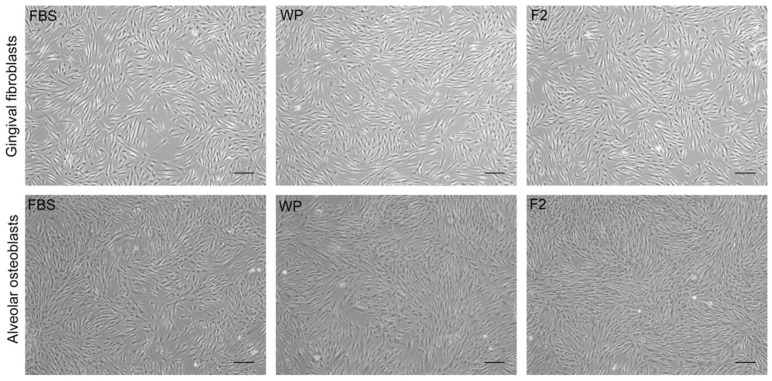
Representative phase contrast images of in vitro cell expansion after the use of PRGF (WP, F2) or FBS as culture medium supplement. Scale bar: 200 µm.

**Figure 2:**
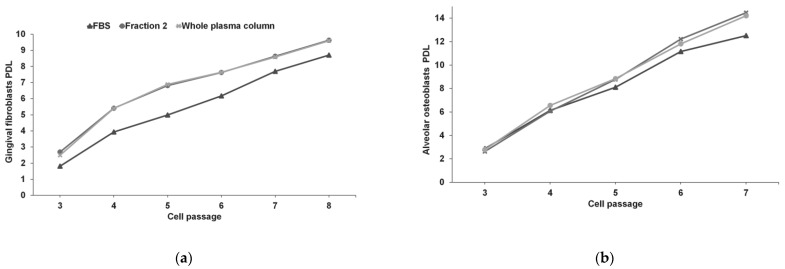
Accumulated PDL throughout the passages after culture with the corresponding supplements. (**a**) Gingival fibroblasts; (**b**) Alveolar osteoblasts.

Isolated gingival and osteoblastic cells were spindle-shaped in their routine culture medium (FBS as culture medium supplement). Culture expansion with F2 or WP did not alter this cell morphology (Figure 1). However, as the number of passages in culture increased, the accumulated PDL was higher for WP and F2 in both phenotypes; so, the proliferative rate of hGFs and hABCs was superior when cells were cultivated with F2 or WP. Alveolar bone cells reached 12.51, 14.47, and 14.21 PDLs in passage 7 after FBS, F2, and WP supplementation for 22 days, respectively. For gingival fibroblasts, the PDLs were 8.7, 9.63, and 9.59 in passage 8 after FBS, F2, and WP supplementation for 43 days, respectively. Figure 2 shows the evolution of PDL for hGFs and hABCs in culture.

### 3.3. Genetic Stability of hGFs and hABCs

Array CGH analysis of cells maintained with FBS, F2 or WP did not reveal the existence of alterations in copy number or genetic instability. The resolution reported in this study was a minimum of 150 Kb for losses and 200 Kb for gains with a minimum of 50 markers. The classification CNV was based on the knowledge and information from CNV databases. The results show that there were no duplications or deletions of any chromosome segment, using hg19 as genomic assembly. The increased proliferation rate in response to Fraction 2 or whole plasma column did not provoke genomic modifications. hGFs and hABCs were genetically stable after 8 and 7 passages respectively, in medium supplemented with PRGF preparations.

## 4. Discussion

Cell-based therapies requires relatively high cell counts that cannot be achieved without ex-vivo cell expansion by performing several passages. Moreover, the production of vaccines and recombinant growth factors will also need ex-vivo cell expansion [45]. The quality of cells is critical and needs to be demonstrated over time. During cell passaging, mutations could occur and alter the homogeneity of cell population. Mutations can promote a selective overtake of small population and end up with a different cell population than the starting one [45]. These changes may lead to different final products of cells, vaccines, or recombinant growth factors. For that, different authorities have established guidelines for bioindustry to guarantee and demonstrate the genetic stability of their biological manufacturing systems.

Moreover, plasma rich in growth factors has been used in different medical fields to enhance tissue healing and patient’s recovery. These includes include oral and maxillofacial surgery, oral medicine, regenerative endodontics, periodontal surgery, orthopaedics and sport medicine, dermatology, and ophthalmology, among others [23,25,42,46,47,48,49,50,51]. For example, plasma rich in growth factors has been applied to manage the clinical symptoms and promote tissue healing in erosive lichen planus and mucous membrane pemphigoid [22,52,53]. Repeated applications of the plasma rich in growth factors have been needed to resolve the symptoms and promote the healing of the soft tissue. In another application, its repeated application in the treatment of medication-related osteonecrosis of the jaw has resulted successful to promote soft tissue healing and bone regeneration [26]. For that, no altering of the genetic stability of the cells is important. In the clinical management of a lesion of erosive lichen planus that is recalcitrant to corticosteroids, other treatments, such as the topical use of tacrolimus or pimecrolimus, have increased the risk of cancer development [54]. No altering of the cells’ genetic stability is thus a critical quality criterion for the use of platelet rich plasma.

To date, foetal bovine serum is the most widely used cell culture supplement for in vitro cell isolation and expansion because of its content in multiple factors capable of supporting growth and proliferation of a variety of cell types and of promoting differential functions [37,38]. For regenerative medicine and cellular therapies, the efforts must be made towards the substitution of any animal-origin additives for culturing human cells. Foetal bovine serum presents potential risk of infections and immunological reactions, limited availability, batch-to-batch variations, and ethical concerns related to animal welfare [34,55,56]. In this sense, during the last decade, human autologous blood derivatives have been established as an alternative to FBS in cell culture [30,31,32,35,37,38,57]. Plasma rich in growth factors is postulated as an interesting substitute in the cell culture media of multiple cellular phenotypes [41,58]. PRGF contains a plethora of autologous proteins and growth factors released upon platelets activation that are key controllers of cell proliferation, differentiation and tissue regeneration, added to the biologically active molecules already present in human plasma [5,46].

The results of this study indicated that the application of plasma rich in growth factors maintained the stability of gingival fibroblasts and alveolar osteoblasts. Plasma rich in growth factors is a leukocyte-free platelet rich plasma with a moderate platelet concentration (2-fold the platelet concentration in the peripheral blood). As derived from human blood, it contains plasma- and platelet-derived biomolecules. The biomolecules equilibrate each other’s action to ensure proper healing and tissue homeostasis. For example, it contains pro-angiogenic factors as well as anti-angiogenic factors. This is an important factor in the safety of plasma rich in growth factors. It has also a positive outcome minimizing the effect of the oxidative stress and reactive oxygen species on the cells, for example, by reducing the DNA double-strand cleavage [59]. Furthermore, it reduces the intensity of inflammation by the inhibition of transactivation of the transcription factor kappaB and the expression of COX2 (cyclooxygenase 2) and CXCR4 C-X-C (chemokine receptor type 4) [18].

Moreover, the effect of two PRGF preparations, Fraction 2, and the whole plasma column has been compared to the gold standard, FBS, on gingival fibroblasts and alveolar osteoblasts proliferation rate and genetic stability. F2 and WP can be employed as cell culture additives without risk of xenogeneic immune reactions or contaminations but increasing the population doubling level of cells in culture. PRGF promoted high cell proliferation without any modification in the cellular morphology or in the chromosomal rearrangement after long-term culture.

There are multiple studies comparing the safety and efficacy of PRP versus FBS as a culture supplement; one interesting aspect is the fact that the in vitro expanded cells should be suitable for clinical applications. In this sense, the culture protocols must comply with good manufacturing practices (GMPs) and regulatory agencies’ standards. Stem cell and PRP therapies arise as the most promising breakthroughs in the treatment of multiple conditions. In general, cells with regenerative potential, e.g., mesenchymal stem/progenitor cells (MSCs), occur in low frequency in tissues and generally have to be propagated to achieve a suitable dose for clinical purpose; however, there must be specific guidelines, standardization and adequate protocols collecting all the parameters involved in the isolation, culture and administration of the ex vivo expanded cells. Moreover, PRP therapies need to be well defined, starting from the obtaining protocol, the type of platelets activation and the dose and schedule of administration, among other parameters [60,61].

Although this preliminary study has certain limitations due to the low number of donors of cells and blood, the CGH analysis performed could help to provide novel data that suggest the use of PRGF supplementation for cell culture. These results would gain strength if they were scaled up to more cell phenotypes and with more blood donors.

In summary, the autologous PRGF technology encompasses all the main advantages of the gold standard FBS as a cell culture supplement and preserves the genomic stability. PRGF arises as a potent safe substitute for human in vitro cell expansion, avoiding the undesired effects of xenogeneic origin products. It has the advantage of providing a 3D-matrix that can act as scaffold for the clinical implantation of primary cells in the organism. Clinically, the repeated application of plasma rich in growth factors would not increase the risk of genetic instability and mutations that may alter the integrity of the tissue.

## 5. Conclusions

PRGF arises as a safe option for repeated applications and as an alternative culture medium supplement for the clinical translation of cell therapy while preserving the genomic stability of cells in vitro.

## Figures and Tables

**Table 1:** Haematological characterization of PRGF plasma preparations. Ratio PRGF/blood means number of platelets in PRGF versus number of platelets in peripherical blood.

	Platelets		Leukocytes		Erythrocytes	
	(×10^3^/µL)	(PRGF/Blood)	(×10^3^/µL)	(PRGF/Blood)	(×10^6^/µL)	(PRGF/Blood)
**Blood**	209		8.2		4.32	
**PRGF-WP**	428	2.05	0.4	0.05	0.03	0.01
**PRGF-F2**	465	2.22	0.3	0.04	0.04	0.01

## Data Availability

All the data are reported in the manuscript.

## Abbreviations

PRGF	Plasma rich in growth factors
FBS	Foetal bovine serum
CGH	Comparative genomic hybridization
PRP	Platelet rich plasma
hGFs	Human gingival fibroblasts
hABCs	Human alveolar osteoblasts
ObM	Osteoblasts culture medium
OGSs	Osteoblast growth supplements
WP	Whole plasma column
F2	Fraction 2
PDL	Population doubling level
D-PBS	Dulbecco’s phosphate buffered saline
CNVs	Copy number variations
COX2	Cyclooxygenase 2
CXCR4 C-X-C	Chemokine receptor type 4
GMPs	Good manufacturing practices
MSCs	Mesenchymal stem/progenitor cells
